# Neurobiological substrates of animal personality and cognition in a nonhuman primate (*Microcebus murinus*)

**DOI:** 10.1002/brb3.1752

**Published:** 2020-07-18

**Authors:** Rebecca Grace Fritz, Elke Zimmermann, Martin Meier, Nadine Mestre‐Francés, Ute Radespiel, Daniel Schmidtke

**Affiliations:** ^1^ Institute of Zoology University of Veterinary Medicine Hannover Hannover Germany; ^2^ ZTL‐Imaging Institute of Laboratory Animal Science Hannover Medical School Hannover Germany; ^3^ MMDN University of Montpellier EPHE INSERM PSL University Montpellier France

**Keywords:** behavior, emotions, executive function, learning, neuroimaging, primates

## Abstract

**Introduction:**

The gray mouse lemur (*Microcebus murinus*) is an important nonhuman primate model in biomedical research. Numerous studies investigated mouse lemur behavior and possible factors underlying interindividual variation in both, animal personality and cognitive performance. Some effects, such as an age‐related decline in executive functioning, have robustly been found across laboratory colonies; however, little is known about the brain structural substrates in mouse lemurs.

**Methods:**

Here, we provide first exploratory data linking in vivo magnetic resonance imaging of 34 mouse lemurs to performance in a standardized, touchscreen‐based task on object discrimination and reversal learning as well as to animal personality under different scenarios in an open field.

**Results:**

High interindividual variability in both brain morphometric and behavioral measurements was found, but only few significant correlations between brain structure and behavior were revealed: Object discrimination learning was linked to the volume of the hippocampus and to temporal lobe thickness, while reversal learning was linked to thalamic volume and the thickness of the anterior cingulate lobe. Emergence latency into the open field correlated with volume of the amygdala. General exploration–avoidance in the empty open‐field arena correlated with thicknesses of the anterior cingulate lobe and fronto‐parietal substructures. Neophilia, assessed as exploration of a novel object placed in the arena, among others, related to the volume of the caudate nucleus.

**Conclusion:**

In summary, our data suggest a prominent role of temporal structures (including the hippocampus) for learning capability, as well as thalamic and anterior cingulate structures for cognitive flexibility and response inhibition. The amygdala, the anterior cingulate lobe, and the caudate nucleus are particularly linked to animal personality in the open‐field setting. These findings are congruent with the comparative psychological literature and provide a valuable basis for future studies elucidating aspects of behavioral variation in this nonhuman primate model.

## Graphical abstract

In mouse lemurs, being valuable animal models in biomedical research, cognition and animal personality have been phenotyped through different behavioral paradigms. Data on underlying neurobiological substrates have been largely missing so far. We show behavioral performance to correlate with region‐specific anatomical measurements similar to the comparative psychological literature described in humans and other mammals, including nonhuman primates.

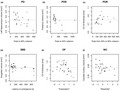

## 1 INTRODUCTION

In biomedical research, meaningful animal models are of high importance in order to allow a good translation of results to human medicine. Being genetically and physiologically closely related to humans, nonhuman primate models, compared to other classical biological models, such as rodents (Lavery, 2000), have gained increasing attention. As a member of the Strepsirrhini primates, the gray mouse lemur (*Microcebus murinus*) is one of the world's smallest primates and, over the past years, has become a valuable animal model, especially in the fields of (brain)aging research and dietetics (Fischer & Austad, 2011; Picq, Villain, Gary, Pifferi, & Dhenain, 2015; Pifferi, Epelbaum, & Aujard, 2019). For example, several aging effects that are known from humans have also been demonstrated in mouse lemurs, including a decrease in motor functions (Némoz‐Bertholet & Aujard, 2003), changes in the endocrine systems (Perret & Aujard, 2005), and in immune functions (Cayetanot, Nygard, Perret, Kristensson, & Aujard, 2009). With regard to cerebral aging, biochemical lesions such as the accumulation of iron (Dhenain et al., 1998), deposits of ß‐amyloid peptide (Bons, Mestre, & Petter, 1992; Mestre‐Frances et al., 2000), and aggregation of Tau protein (Delacourte et al., 1995) have been described to naturally develop in aging mouse lemurs. Furthermore, different patterns of brain atrophy, such as ventricular expansion, region‐specific volumetric decline, and cortical white matter shrinkage, were found (Dhenain, Chenu, Hisley, Aujard, & Volk, 2003; Fritz et al., 2020; Kraska et al., 2011; Picq, Aujard, Volk, & Dhenain, 2012; Sawiak, Picq, & Dhenain, 2014). Regarding dietetics, the effects of long‐term caloric restriction and food supplementation, for example with resveratrol (Dal‐Pan, Pifferi, Marchal, Picq, & Aujard, 2011) and omega‐3 polyunsaturated fatty acids (Languille, Aujard, & Pifferi, 2012; Royo et al., 2018; Vinot et al., 2011) have been investigated in mouse lemurs.

In many of the abovementioned studies, a central research question was whether age or dietary aspects are linked to behavioral variation, including variations in cognitive performance and/or animal personality. For example, one approach used a test battery originally designed for mouse lemurs and described age‐dependent decline in executive functions, such as set shifting and pairwise spatial as well as visual discrimination reversal learning (Picq, 2007; Picq et al., 2012). Another approach, in which more standardized testing procedures for the comparative quantification of cognition were used (e.g., Joly, Ammersdörfer, Schmidtke, & Zimmermann, 2014; Schmidtke, Ammersdörfer, Joly, & Zimmermann, 2018), confirmed an age‐dependent loss in cognitive flexibility during reversal learning and additionally found object discrimination acquisition to be affected in aged mouse lemurs (Joly et al., 2014). Recent dietary studies found a beneficial effect of long‐term caloric restriction and resveratrol (Dal‐Pan et al., 2011) or omega‐3 polyunsaturated fatty acids supplementation (Vinot et al., 2011) on spatial memory performance in mouse lemurs, suggesting nutritional history to contribute to natural, phenotypic variation in cognition.

The classical testing environment for the quantification of animal personality‐related behavior in small animals is the open‐field maze (Walsh & Cummins, 1976), which was originally developed for the assessment of motivation in rats (Hall & Ballachey, 1932). In this setting, measurements of locomotor activity are used to quantify animal personality traits, ranging from shyness–boldness and exploratory behavior to risk‐taking behavior and anxiety, including fear of novelty or open spaces (Marks, 1987; Walsh & Cummins, 1976). Open‐field maze‐based studies in mouse lemurs have investigated various locomotor behaviors (Dal‐Pan et al., 2011; Némoz‐Bertholet & Aujard, 2003) as well as different personality traits (Dammhahn, 2012; Vinot et al., 2011) and, as mentioned above, detected age‐related, diet‐related, and sex‐specific variations in activity, exploration, and anxiety.

Despite the fact that mouse lemur phenotypic variation in brain structure and behavior are well documented and have robustly been demonstrated across setups and laboratory populations (see above), little is known about how they are linked. Especially in studies on mouse lemur cognition, authors often speculate upon neuroanatomical substrates for different cognitive functions (Joly et al., 2014; Picq, 2007; Trouche, Maurice, Rouland, Verdier, & Mestre‐Francés, 2010). Speculations are usually based on what is known from humans and/or lesioning studies in rodent models, but data from mouse lemurs supporting these speculations are largely missing due to ethical principles concerning invasive research in primates. The only study directly linking specific brain structures to cognition is an in vivo structural brain MRI study, describing executive functioning to be related to volume of the septal region, the caudate nucleus and the splenium, as well as to cingulate cortices (Picq et al., 2012). Therefore, the aim of this study was to further explore possible relationships between brain structure and cognitive and animal personality‐related behavioral measurements. We correlated available in vivo MRI‐derived morphometric data from our breeding colony with behavioral data of the same subjects from standardized cognitive tests on pairwise visual discrimination learning and its reversal and with data from open‐field‐based behavioral testing procedures.

## 2 MATERIAL AND METHODS

### 2.1 Ethical statement

From a breeding colony of the Institute of Zoology of the University of Veterinary Medicine in Hannover (LAVES; reference number: AZ 42500/1H (breeding and maintenance)), Germany, 34 adult mouse lemurs (*Microcebus murinus*) were involved in this study. All here‐reported experiments were performed in compliance with the German Animal Welfare Act, the NRC Guide for the Care and Use of Laboratory Animals, and the Directive 2010/63/EU of the European Parliament on the protection of animals used for scientific purposes. They were approved by the Animal Welfare Committee of the University of Veterinary Medicine and licensed by the Lower Saxony State Office for Consumer Protection and Food Safety (LAVES; reference numbers: AZ 33.19‐42502‐05‐11A116 (MRI), AZ 33.9‐42502‐05‐10A080 & AZ 33.12‐42502‐04‐14/1454 (cognitive/ behavioral experiments).

### 2.2 Subjects

In vivo MRI scanning was conducted on all 34 individuals (18♀♀/16♂♂, age range: 3.1 to 11.9 years). 21 animals (12♀♀/9♂♂) of this total sample were additionally involved in cognitive testing (see Section 2.4) and 30 animals (14♀♀/16♂♂) of the total sample took part in open field‐based experiments (see Section 2.5). Due to the logistic effort of in vivo MRI in primates, MRI scans could not be performed directly after the behavioral experiments, resulting in different delays between cognitive/behavioral testing and MRI (cognitive testing: min = 1.85 years, max = 2.89 years, mean = 2.42 years; open field‐based testing: min = 0.82 years, max = 3.86 years, mean = 2.40 years). Mathematical procedures used to correct for this delay are described in the “statistical analyses” section below.

Subjects commonly lived in small same‐sex groups of two to four members. Temperature (23–25°C) and relative humidity (50%–60%) were kept constant. Cages were equipped with climbing opportunities as environmental enrichment and one or two wooden boxes per individual to provide shelter. The diet of the mouse lemurs changed on a daily basis between seasonal fresh fruit mixed with vegetables and banana mash (Milupa Nutricia GmbH; Bad Homburg v. d. H., Germany) enriched with vitamins and minerals. Mealworms and locusts were offered weekly as additional protein source (for details on the diet see Hülskötter et al., 2017). To compensate the additional caloric intake from the food reward, each subject's regular diet was slightly reduced during cognitive testing. Animals lived under a seasonally fluctuating, reversed light cycle, with a long‐day period (LD 14:10) of 8 months and a short‐day period (LD 10:14) of 4 months. All cognitive/behavioral experiments started during the long‐day periods. Prior to the experiments, subjects were checked for good health and for eye diseases (Dubicanac et al., 2016, 2017) by a veterinarian, as some of the experimental procedures depended on visual information processing. All tested subjects were *naïve* to the touchscreen‐based cognitive tests and to the open‐field maze.

### 2.3 Structural brain analyses

For brain morphometry, three‐dimensional T2‐weighted MRI was performed in vivo, under general anesthesia (for further details see Kästner, Tünsmeyer, & Schütter, 2016). Body temperature was monitored and regulated with a heating pad (Bruker T10964) at a constant level (±1°C). Heart rate and respiratory rate were constantly monitored on a magnetic resonance‐compatible physiological monitoring system (SA Instruments, Stony Brook, NY, Model 1,030) to ensure the animal's stability. Scans were conducted at the Imaging Center of the Institute of Laboratory Animal Science of Hannover Medical School, with a Bruker 7T Pharmascan (70/16 Bruker BioSpin MRI GmbH, Ettlingen, Germany) equipped with a high performance gradient system with 300 mT/m maximum gradient amplitude and 0.35 ms rise time. A combination of RF RES 300 1H 089/072 QUAD TO AD and RF ARR 300 1H M. HRT. RO AD AUTOPAC (Bruker BioSpin MRI GmbH) coils was used for all scans. Images were acquired using rapid acquisition with relaxation enhancement (RARE) sequences at the following parameters: repetition time = 2,500 ms, effective echo time = 11.6 ms, field of view = 3 × 3 × 3 cm, acquisition matrix = 128 × 128 × 128, reconstruction matrix = 256 × 256 × 256, resolution = 234 μm, bandwidth = 25 kHz, and flip angle = 113.8°.

MRI images of all 34 subjects were preprocessed according to previously published protocols (Picq et al., 2012; Sawiak et al., 2014) to ensure spatial homogeneity and to secure interindividual comparability. Morphometric measurements were taken manually and in two steps: Based on regions of interest (ROIs), six different brain areas (thalamus, splenium of the corpus callosum, septal region, caudate nucleus, hippocampus, and amygdala) were volumetrically measured and normalized by each subject's intracranial volume. For a detailed description of the ROI measurements, see Picq et al., 2012. In addition, thickness of the cerebral cortex was measured at 25 reference positions in different brain areas and summarized according to the respective brain lobe (compare Sawiak et al., 2014). Cortical thickness measurements are also presented as normalized values corrected against the intracranial volume.

### 2.4 Cognitive phenotyping

**FIGURE 1:**
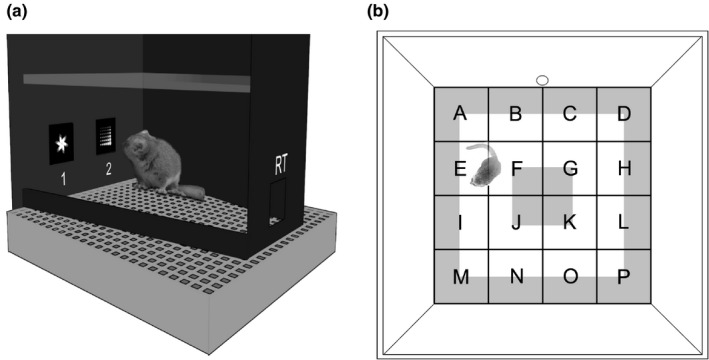
Experimental setups. (a) Schematic drawing of the trapezoid automated touchscreen setup used for cognitive testing. The touchscreen was located at the long base of the chamber. The animals could access the touchscreen through two response windows (1 + 2), in which the visual stimuli were presented. Through a reward tray (RT), correct responses were rewarded with 25 μl of apple juice. (b) Schematic drawing of the open‐field arena consisting of a square floor plate (76 × 76 cm), surrounded by walls of 40 cm height. The floor was virtually segmented into 16 equally sized zones (A‐P; 19 × 19 cm each), a “central zone” (inner gray square; 19 × 19 cm) and a “periphery” (outer gray area; width: 9.5 cm). For the novel object test, an ellipsoid stone was placed in the center of the arena. Subjects could enter the arena directly from their sleeping box through a circular hole in one of the wall panels (here top panel between “b” and “c”)

Cognitive phenotypes were determined for 21 animals that were part of a previous and larger study on age‐related cognitive decline in mouse lemurs (Joly et al., 2014). Phenotypes were assessed using a customized version of the Bussey‐Saksida Touchscreen Chamber (Model 80,604, Campden Instruments LTD; for a schematic drawing see Figure 1a). In short, individual object discrimination and associative learning performance as well as cognitive flexibility were quantified through a touchscreen‐based, standardized visual pairwise‐discrimination (PD) and pairwise‐discrimination reversal (PDR) learning paradigm. Subjects were tested in one session per day (with 30 trials per session) to learn to discriminate between two simultaneously presented visual stimuli and to respond to one of them (chosen to be the rewarded stimulus) by touching the screen with their hand or nose to receive a reward (25 µl of apple juice for each correct choice). During the PD acquisition, subjects were trained to reach a criterion of 80% (later on referred to as PD 80) or more correct choices in two consecutive sessions to quantify individual object discrimination learning performance (e.g., Winters, Bartko, Saksida, & Bussey, 2010). Once this criterion was reached, the stimulus‐reward contingency was reversed in subsequent sessions (PDR). For the PDR, two criteria were defined. Firstly, the number of trials each individual needed to reach a performance of 50% or more correct choices in two consecutive sessions (later on referred to as PDR 50) was measured. This criterion was used to quantify the subject's cognitive flexibility (Graybeal et al., 2011). Afterward and secondly, the number of trials each individual needed to rereach a criterion of 80% (later on referred to as PDR 50–80) or more correct choices in two consecutive sessions was used to assess the formation of stimulus‐reward habits without object discrimination learning (Graybeal et al., 2011). Transport of the respective experimental animal from its home cage to the testing chamber and back took place in the subject's individual sleeping box, from which it could directly be released into the chamber without visual contact to the experimenter. A more detailed description of pairwise‐discrimination learning and its reversal in mouse lemurs including the pretraining protocol and details on the test chamber is available in (Joly et al., 2014).

### 2.5 Behavioral phenotyping

To evaluate each individual's “personality,” 30 subjects were tested in a standard open‐field (OF) test and in open‐field‐based novel object (NO) and sleeping box emergence (SBE) tests. The open field arena consisted of a square floor plate (76x76 cm), surrounded by 40 cm high walls. It was located in an echo‐reduced room and four red light bulbs installed in the corners behind the walls of the arena provided dim (~1 lux) homogeneous illumination during the experiments. To start an experiment, the subject's sleeping box, in which it was also transported to the setup, was positioned next to the arena. Similar to the cognitive testing, the animal could directly enter the arena through a hole in one of the walls without direct contact to the experimenter. Experiments were videotaped from above (camera: SuperSteadyShot DCR‐SR210, SONY Corporation; operated in NightShot mode). Offline frame‐by‐frame video analysis was later performed using The Observer XT 10 (The Observer 10.5.572, Noldus Information Technology, 1990–2011). For analyses, the arena floor was virtually segmented into 16 equally sized zones (A‐P; 19x19 cm each), a periphery (reaching 9.5 cm from the walls into the floor area), and a square central zone (19x19 cm around the center; Figure 1b).

In the first open‐field‐based experiment, the SBE, the latency from the beginning of the test session to the subject's emergence from its sleeping box into the open field arena (both hands and feet are within the arena) was measured as the sole variable to quantify “shyness” (e.g., Brown, Jones, & Braithwaite, 2005). If a subject did not enter the arena during a 15 min time limit, the latency was set to 900 s. After a given subject had emerged or the time limit was reached, the session was ended and the animal was transported back to its home cage.

For the second open‐field‐based experiment, the OF, each subject could freely explore the arena for 15 min after it had left the sleeping box. During that time, the door to the sleeping box remained closed. Measurements taken during subsequent analyses included the total number of visited zones (A‐P) as well as the number of zone changes, the number of times the subject straightened up, the duration a subject spent with freezing, walking/running, or climbing, respectively, the number of times the subject jumped, the total duration the subject spent in the central zone, the periphery, and the emergence zone, the number of times the central zone was entered, and the latency from the beginning of the test session until the individual entered the central zone for the first time (with both hands). Latency was set to 900 s if the subject did not enter the central zone.

For the third open‐field‐based experiment, the NO, an ellipsoid stone (volume: 30 cm^2^) was placed in the center of the arena and the subject was allowed to explore the arena under the same conditions as for the OF (i.e., 15 min of free exploration, locked sleeping box). Here, the following parameters were measured: The latency from the beginning of the session to the subject's first approach toward the object (i.e., entering the center zone) and to the subject's first physical contact (nose or hand) with the object, the frequency of approaches, as well as the frequency of physical contacts, the total duration of contacts and the number of times the object was being displaced. If the subject did not approach the object or interact with it during the 15 min duration of the session, the respective latency was set to 900 s.

### 2.6 Statistical analyses

Data analysis was performed using R (R Core Team, 2019). Since many of the analyzed variables were not normally distributed (Shapiro–Wilk test; shapiro.test‐function in R), two‐tailed Spearman correlation analyses (cor.test‐function in R; method = “spearman”) were used to explore potential links between brain morphometry and behavior. To reduce the number of variables from the open field‐based experiments used for correlation analyses with MRI measurements, that is, to obtain one representative variable per test (OF and NO), principal component analyses (PCA) were conducted (psych‐package in R). Overall measures of sample adequacy were 0.57 for the OF variables and 0.56 for the NO variables. Item MSA varied between 0.31 and 0.74.

To account for the different delays between the cognitive/ behavioral experiments and MRI, (compare supporting materials Table S1), correlation analyses were conducted twice: (a) with the morphological raw data (actually measured values) and (b) with morphological data corrected for the variable delay using predictions for age‐related changes of the different measurements from sex‐specific regression models obtained from a larger MRI data set from our colony (Fritz et al., 2020; for regression estimates see Table S2). The main text reports result from the delay‐corrected analyses. Results from the uncorrected analyses are only reported, if both analyses are in disagreement. In most cases, however, results from both of these analyses matched, suggesting that interindividual variance in brain morphology for most variables was higher than potential structural changes expected to occur during the delay. For direct comparison, results from the raw data analyses are presented in the supporting materials (Tables S5 and S6).

## 3 RESULTS

**FIGURE 2:**
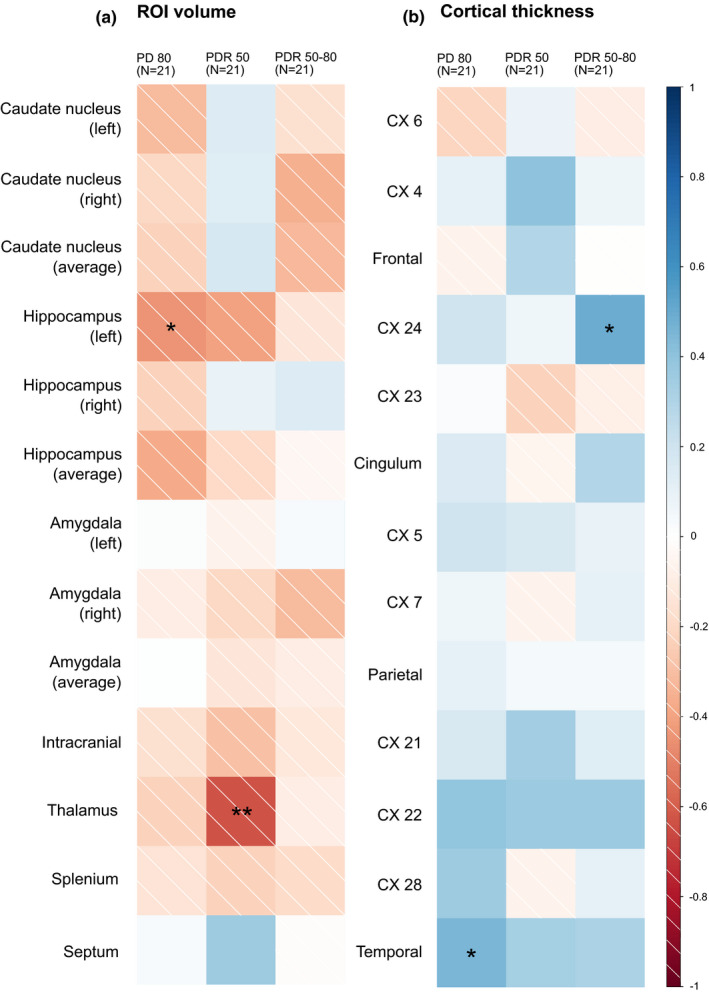
Graphical summary of the correlations between brain morphometry and cognition (*N* = 21). (a) ROI volume‐related analyses; (b) cortical thickness‐related analyses. (a, b) From left to right: PD 80 (object discrimination learning), PDR 50 (early reversal learning), and PDR 50–80 (late reversal learning); individual squares represent the results of a single correlation analysis (Spearman's). Strength and direction of the correlation are color‐coded according to the legend next to b. Significant correlations are marked with asterisks (significance code: *p ≤ .05; **p ≤ .01)

**FIGURE 3:**
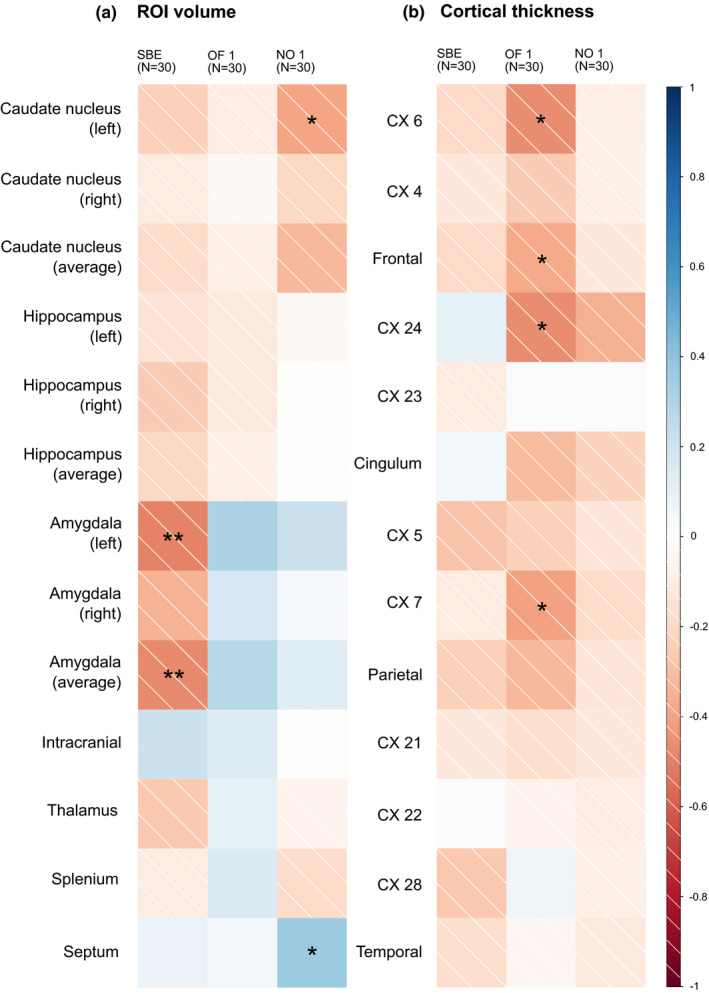
Graphical summary of the correlations between brain morphometry and personality (*N* = 30). (a) ROI volume‐related analyses; (b) cortical thickness‐related analyses. a, b From left to right: SBE (“shyness”), OF 1 (“exploration”), and NO 1 (“neophilia”); individual squares represent the results of a single correlation analysis (Spearman's). Strength and direction of the correlation are color‐coded according to the legend next to b. Significant correlations are marked with asterisks (significance code: *p ≤ .05; **p ≤ .01)

In general, all assessed variables, both morphometric and behavioral, showed high interindividual variability and correlations between morphometric measurements and behavioral measurements were quite rare given the number of possible relations explored (see below and Figures 2 and 3).

### 3.1 Structural MRI and cognitive phenotyping

#### 3.1.1 PD 80

**FIGURE 4:**
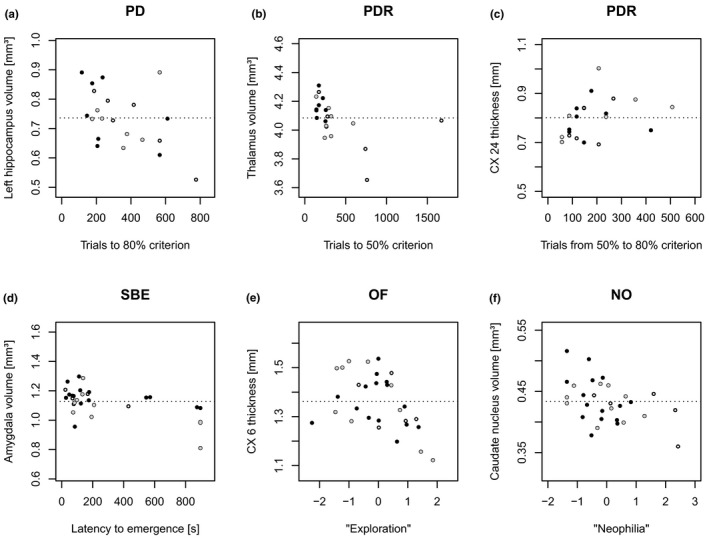
Graphs of exemplary correlations between brain morphometry and cognitive and behavioral measurements. (a–c) Cognitive phenotyping (*N*_♀♂ _= 21), (d–f) behavioral phenotyping (*N*_♀♂_ = 30). (a) PD 80 (object discrimination learning) versus volume of the left hippocampus; (b) PDR 50 (early reversal learning) versus thalamic volume; (c) PDR 50–80 (late reversal learning) versus cortical thickness of CX 24 (cingulate lobe); (d) SBE (“shyness”) versus volume of the amygdala; (e) OF 1 (“exploration”) versus cortical thickness of CX 6 (frontal lobe); (f) NO 1 (“neophilia”) versus volume of the caudate nucleus. (a‐f) Filled black circles represent young subjects (age at test ≤ 3 years), filled gray circles represent middle aged subjects (age at test > 3 and ≤ 5 years), hollow circles represent old subjects (age at test > 5 years; age classification is in line with Marchal et al., 2012), and horizontal dashed lines represent the age‐independent sample mean of the respective brain structural measurement

For the pairwise discrimination acquisition (PD 80), a significant negative correlation was found between the volume of the left hippocampus and the number of trials to criterion (*N* = 21, *r*_SP_ = −0.44, *p* = .045; Figures 2a and 4a) as well as the cortical thickness of the temporal lobe and the number of trials to criterion (*N* = 21, *r*_SP_ = 0.45, *p* = .04; Figure 2b), but only in the delay‐corrected analysis.

#### 3.1.2 PDR 50

For the early phase of the reversal learning (PDR 50), negative correlations of brain str ucture volume with trials to criterion were found for the volume of the thalamus (*N* = 21, *r*_SP_ = −0.64, *p* = .002; Figures 2a & 4b).

#### 3.1.3 PDR 50–80

For the late phase of the reversal learning (PDR 50–80), cortical thickness of CX 24 (cingulate lobe) positively correlated with trials to criterion (*N* = 21, r_SP_ = 0.49, *p* = .023; Figures 2b and 4c). For an overview of all correlation analyses performed (brain morphometry versus cognition), see supporting material Tables S3 and S5.

### 3.2 Structural MRI and behavioral phenotyping

By submitting open‐field maze‐based behavioral data to principal component analyses, components with eigenvalues greater than one (Kaiser–Guttman Rule, (Kaiser, 1991)) were revealed for both tests, the OF and the NO. OF 1 showed high factor loadings (>0.75) on all activity and exploration related variables. For readability, OF 1 will subsequently be called “exploration.” This component explains 45% of the variability within the OF data (Table 1). For the NO, high factor loadings (>0.77) on all object‐related variables, that is, measurements of exploration and neophilia, were found for NO 1. Thus, NO 1 will subsequently be called “neophilia.” This component accounts for 66% of the variation within the NO data (Table 1). For the SBE, only one variable was measured (latency to emergence), which was directly used for correlation analyses.

**TABLE 1:** Summary of the principal component analyses

Variable	OF 1: “exploration”	Variable	NO 1: “neophilia”
Frequency of zone changes (A‐P)	**0.88**	Latency to first contact with the NO [s]	**−0.80**
Number of visited zones (A‐P)	0.68	Number of object displacements	0.65
Duration walking/running [s]	**0.86**	Latency to approach the NO [s]	**−0.77**
Number the subject straightened up	0.66	Frequency of approaches toward the NO	**0.87**
Duration of freezing [s]	−0.36	Frequency of contacts with the NO	**0.91**
Number the subject jumped	0.16	Duration of contacts with the NO [s]	**0.83**
Duration of climbing [s]	0.21		
Duration spent in the emergence zone [s]	−0.04		
Duration spent in the central zone [s]	**0.75**		
Duration spent in the periphery [s]	0.10		
Frequency of central zone entries	**0.89**		
Latency to first entry of the central zone [s]	**−0.80**		
Eigenvalue	4.63		3.95
Var. Expl.	45%		66%

Note

Components with eigenvalues greater than one were revealed for both behavioral tests, the OF and the NO. Bold numbers indicate factor loadings higher than 0.7.

#### 3.2.1 SBE

Correlation analyses for the SBE revealed a significant decrease in volume of the left amygdala and the averaged amygdala volume with increased latency to emergence (= “shyness”; *N* = 30, *r*_SP_ ≤ −0.48, *p* ≤ .008; Figures 3a and 4d). The individual with the highest latency to emergence (878 s) and three subjects, which did not leave the sleeping box at all (latency to emergence = 900 s), all presented with amygdala volumes that were below the sample mean (1.13 mm^3^; Figure 4d).

##### OF—Principal component 1 (“exploration”)

The “exploration” component (OF1) correlated negatively with thickness of CX 6 of the frontal lobe (*N* = 30, *r*_SP_ = −0.47, *p* = .01; Figures 3b and 4e) and thickness of CX 24 of the cingulate lobe (*N* = 30, *r*_SP_ = −0.47, *p* = .01; Figure 3b). The same was true for the thickness of the frontal lobe average and parietal subregion CX 7 (*N* = 30, *r*_SP_ ≤ −0.37, *p* ≤ .043; Figure 3b; compare Table S5), but only in the delay‐corrected analysis.

##### NO—Principal component 1 (“neophilia”)

The “neophilia” component (NO1) negatively correlated with the volume of the left caudate nucleus (*N* = 30, *r*_SP _= −0.40, *p* = .029; Figures 3a and 4f). The septal volume correlated positively with “neophilia” (*N* = 30, *r*_SP_ = 0.37, *p* = .045; Figure 3a; compare Table S6), but only in the delay‐corrected analyses. For an overview of all correlation analyses, see supporting material Tables S4 and S5.

## 4 DISCUSSION

Numerous studies have investigated possible determinants of interindividual behavioral variation in mouse lemurs. Often, age‐related and/or dietary aspects have been explored and could be linked to variations in both, individual cognitive performance (e.g., Joly et al., 2014; Picq et al., 2012) and/or individual behavioral characteristics in tests of animal personality, such as the open‐field test (e.g., Dammhahn, 2012; Vinot et al., 2011). In addition, behavioral phenotypes in mouse lemurs have been linked to genetics (e.g., Zablocki‐Thomas, Herrel, Karanewsky, Aujard, & Pouydebat, 2019). Given that animal behavior is ultimately controlled by the brain, it is reasonable to assume that all of the aforementioned factors are linked to cognition and personality via brain morphology and/or region‐specific cytoarchitecture and physiology. However, to date, little is still known about the neurobiological substrates of different psychological constructs in mouse lemurs and whether they accord to neurobiological substrates in humans or other well‐established animal models, such as rodents. This is partially due to the fact that invasive research in primates, for ethical reasons, is only justifiable as the ultima ratio. The here‐presented exploratory analyses of noninvasive MRI with standardized behavioral data, even though coming with the downside of decreased anatomical precision and lack of causality compared to invasive (e.g., lesioning of pharmacological) studies, provide first insights to which brain areas may be important for the different constructs that were assessed. In summary, our data suggest a role of temporal structures for learning capability, cingulate and thalamic structures for cognitive flexibility and response inhibition, as well as linkage of the amygdala, the caudate nucleus, and the cingulate lobe to animal personality. These results are in line with the only comparable study in mouse lemurs and largely match data from humans, as will be discussed in more detail in the following paragraphs.

### 4.1 Cognition

In humans, early hypotheses about the functional parcellation of the brain were usually based on patient data, such as the clinical cases of aphasia described by Broca of the famous case of Phineas Gage (see Van Horn et al., 2012; for a recent discussion of that case). With the advent of in vivo imaging techniques, additional proof could be collected from larger samples of healthy individuals. For example, using structural MRI it was shown that London taxi drivers with high navigational experience had larger volumes of the posterior hippocampus compared to a control group, supporting the idea of a prominent role of the hippocampus in navigation (Maguire et al., 2000). This role of the human hippocampus and adjacent areas was supported by functional MRI data shortly thereafter (Hartley, Maguire, Spiers, & Burgess, 2003; for a recent review see Epstein, Patai, Julian, & Spiers, 2017). While functional MRI today is extensively used to further explore functional parcellation and connectivity in humans, in small animals, such as mouse lemurs, functional MRI remains methodologically challenging and needs further development.

The cognitive constructs addressed here, that is, procedural object discrimination and stimulus‐reward associative learning as well as response inhibition/cognitive flexibility were quantified using a highly standardized, computerized task on visual pairwise discrimination learning and its reversal. To give a complete overview of the neurobiological bases of learning and memory is beyond the scope of this discussion. In brief, based on studies from humans and nonhuman primates, the current opinion on the neurobiological substrates for the cognitive functions considered here is the following: The hippocampus and surrounding medial temporal areas play a prominent role in spatial learning and cognition as well as in the encoding of contextual/episodic memory, long‐term memory consolidation, and object memory and recognition (Bachevalier, 2019; Lisman et al., 2017). Procedural learning and memory, on the other hand, are strongly dependent on the cerebellum and subcortical structures, such as the basal ganglia (Foerde & Shohamy, 2011). Executive functions, which allow for a flexible adaptation to changing environmental conditions and include response inhibition and cognitive flexibility, predominantly rely on prefrontal circuitry (Robbins, 1996). Due to the procedural and nonspatial nature of the pairwise discrimination task we used for the quantification of object discrimination and reversal learning in our subjects, it is usually considered to be largely independent of the hippocampus and other medial temporal structures, but to rather rely on striatal structures (e.g., Bussey et al., 2012; Teng, Stefanacci, Squire, & Zola, 2000). However, correlations were found between hippocampal volume as well as the thickness of the temporal lobe and PD performance. This suggests that these structures do play a role in task acquisition, probably through their involvement in object identification and recognition memory (e.g., Baxter & Murray, 2001; Cohen et al., 2013; James, von Oertzen, Norbury, Huppertz, & Brandt, 2018; de Lima, Luft, Roesler, & Schroder, 2006; Winters et al., 2010).

For the early phase of the reversal test, which places high demands on response inhibition and cognitive flexibility, our data strongly suggest an involvement of thalamic structures. Individual performance in the late phase, on the other hand, relates to anterior cingulate morphology. Again, these findings are in line with literature from humans and animal models: The thalamus and cingulate regions (both anterior and posterior) have been linked to response inhibition (e.g., Chudasama, Bussey, & Muir, 2001; Förstl & Sahakian, 1993) and behavioral flexibility in response to changes in environmental contingency (e.g., Pearson, Heilbronner, Barack, Hayden, & Platt, 2011; Walton, Croxson, Behrens, Kennerley, & Rushworth, 2007), respectively. Interestingly, our data also support the only previously published study correlating structural brain measurements to cognitive ability in mouse lemurs (Picq et al., 2012). This study also found significant correlations between executive functioning (assessed as a composite score of set shifting and reversal learning) and anterior and posterior cingulate thickness. Furthermore, spatial memory performance in the same study was linked to hippocampal volume and thickness of the entorhinal cortex (Picq et al., 2012).

### 4.2 Animal personality

The concept of animal personality acknowledges that individuals of a given species, subpopulation, or even genetically identical laboratory strains show consistent (i.e., repeatedly measurable) interindividual differences in their behavior (Réale, Reader, Sol, McDougall, & Dingemanse, 2007). For mouse lemurs, the temporal stability of interindividual behavioral differences in the here‐used, open‐field‐based experiments has been confirmed numerous times, both in the field and under laboratory conditions (Dammhahn, 2012; Verdolin & Harper, 2013; Zablocki‐Thomas et al., 2018; Zablocki‐Thomas et al., 2019). In our own colony, the repeatability, as estimated using repetition experiments of the SBE, OF, and NO and the calculated correlations (Spearman) of individual scores between first and second repetition, is high for the latency to emerge (SBE; *N* = 47, *r*_SP_ = 0.72, *p* < .001) and exploration (OF; *N* = 47, *r*_SP_ = 0.75, *p* < .001) and a little lower for neophilia (NO; *N* = 47, *r*_SP_ = 0.62, *p* < .001). To differentiate between relevant personality traits, one of the most‐used conceptual frameworks of animal personality was established by Réale and colleagues (Réale et al., 2007). In their article, five traits were distinguished and defined, namely shyness–boldness, exploration–avoidance, activity, aggressiveness, and sociability. In this conceptual context, the standard open‐field test (OF) primarily quantifies individuality on an exploration–avoidance continuum. In experimental, biomedical research in animals, anxiety has been quantified (Seibenhener & Wooten, 2015) with a positive association of anxiousness to avoidance. By adding an unknown object to the open‐field arena (NO), individual neophilia can additionally be assessed. The SBE used in our study is not explicitly mentioned in the article of Réale and colleagues, but is routinely used in different studies to quantify individuality on a shyness–boldness continuum (e.g., Brown et al., 2005).

The amygdala, as a major component of the limbic system, has often been investigated in human literature in the context of personality research (Davidson, 2003; Roxo, Franceschini, Zubaran, Kleber, & Sander, 2011) and is described to be mainly involved in emotional modulation and information processing between prefrontal and temporal association cortices (Sergerie, Chochol, & Armony, 2008). Furthermore, studies also showed high involvement of the amygdala when responding to stimulus novelty (Weierich, Wright, Negreira, Dickerson, & Barrett, 2010). Finally, in open‐field experiments in rodents, the amygdala has been shown to convey location‐modulated (corner versus center) information and to likely code for changes in the exploratory state of the animal (Gründemann et al., 2019). In our findings, the amygdala strongly related to the subject's first emergence into the open‐field maze. Individuals with small amygdala volumes showed high latencies to emerge from their shelter. Similarly, in a study in macaques that were classified as either bold or reserved, based on the time they spent in the unprotected area of a play room, it was found that bold animals presented with bigger amygdalae as compared to reserved conspecifics (Haley et al., 2012). Additionally, we also found correlations between exploration during the OF and both, the premotor area (CX 6) of the frontal cortex and the visuomotor region (CX 7) of the parietal cortex, which are involved in the planning and execution of complex, coordinated movements (Averbeck & Seo, 2008; Towe & Luschei, 1981; Weinrich, Wise, & Mauritz, 1984). Finally, our data suggest a link between the anterior cingulate cortex (CX 24) and exploration in the OF. The anterior cingulate cortex has previously been investigated in different studies to correlate with novelty (Gardini, Cloninger, & Venneri, 2009), which matches our results, as subjects were *naïve* to the open field arena during the OF, which means the subjects were confronted with a new, unknown environment. In the NO, neophilia related to the volume of the caudate nucleus and the septal region. The caudate nucleus, as described before, is involved in procedural reward learning and memory functions (Grahn, Parkinson, & Owen, 2009), but is also considered to integrate spatial information with motor processes for the initialization and execution of directed movements (Simon et al., 2002; Villablanca, 2010). Therefore, it has further been suggested to be involved in both, curiosity and goal‐directed responses to novel stimuli in the environment (e.g., Cigrang, Vogel, & Misslin, 1986; Kang et al., 2009). In line with this, striatal lesioning in mice was found to increase the number of physical interactions with a novel object in an NO (Cigrang et al., 1986).

## 5 CONCLUSION

As discussed in the previous paragraphs, our findings of a first exploratory linkage of brain morphology to behavior are in line with data on brain structural substrates of different behavioral performances in humans as well as in other primate and nonprimate animal models. Furthermore, they confirm a prominent role of the mouse lemur's cingulum in executive control, as previously suggested (Picq et al., 2012). For open‐field‐based testing, which is widely used in mouse lemurs to quantify both, personality traits and anxiousness (e.g., Dammhahn, 2012; Vinot et al., 2011; Zablocki‐Thomas et al., 2019), our findings suggest limbic structures (especially the amygdala and cingulate regions), involved in emotional processing, as well as the caudate nucleus to underlie individual, phenotypic variation in the open‐field maze. Therefore, our study provides likely candidates for neurobiological substrates of interindividual variation in both, cognition and animal personality in mouse lemurs and a valuable new basis for future studies on comparative psychology in this important nonhuman primate model.

## CONFLICT OF INTEREST

None declared.

## AUTHOR CONTRIBUTIONS

Rebecca G. Fritz involved in validation, formal analysis, investigation, writing–original draft, writing–review and editing, and visualization; Elke Zimmermann involved in conceptualization, resources, project administration, and funding acquisition; Martin Meier involved in methodology, investigation, resources, and writing–review and editing; Nadine Mestre‐Francés involved in conceptualization, writing–review and editing, project administration, and funding acquisition; Ute Radespiel involved in writing–original draft, writing–review and editing, and supervision; Daniel Schmidtke involved in conceptualization, formal analysis, investigation, writing–original draft, writing–review and editing, supervision, project administration, and funding acquisition.

### Peer Review

The peer review history for this article is available at https://publons.com/publon/10.1002/brb3.1752.

## Supporting information

Appendix S1

## Data Availability

The data that support the findings of this study are available from the corresponding author upon reasonable request.
