# Supplementary material for: Neurobiological substrates of animal personality and cognition in a nonhuman primate (Microcebus murinus)
Source: Brain Behav. 2020 Jul 18;10(9):e01752. doi: 10.1002/brb3.1752 (PMC7507526; doi:10.1002/brb3.1752)
Supplement: Supplementary file 1 — Appendix S1 [file BRB3-10-e01752-s001.pdf]

Tab. S1: Behavioral performance variables for each tested animal. The table provides animal-ID, sex, delay between cognitive testing and MRI [years], number of trials to reach the criterion for the PD 80, the PDR 50, and the PDR 50-80, delay between open-field based testing and MRI [years], latency to emergence for the SBE [s], as well as exploration (first principal component from the OF) and neophilia (first principal component from the NO) values.

Animal ID	Sex	Delay Cognition	PD 80	PDR 50	PDR 50-80	Delay OF	SBE	OF 1	NO 1
1	f	1.97	780	1680	120	0.82	74	1.31	2.35
2	f	2.26	270	150	150	1.05	82	0.47	2.45
3	f	2.44	360	600	360	1.36	197	0.46	-0.19
4	f	1.88	120	229	90	0.90	42	0.31	-1.33
5	f	1.98	180	330	60	1.11	78	-0.33	0.08
6	f	2.25	210	300	240	1.44	87	1.47	1.31
7	f	2.69	237	148	60	1.69	213	1.88	-1.09
8	f	2.35	215	261	120	1.53	74	0.91	-0.58
9	f	2.34	191	285	90				
10	f	2.41	300	180	150				
11	f	2.29	570	769	210				
12	f	2.11	180	155	90				
13	f					1.40	102	0.76	0.60
14	f					1.78	900	-1.20	0.18
15	f					1.70	900	-1.44	-0.29
16	f					1.27	142	-0.98	0.67
17	f					1.46	878	0.66	0.39
18	f					1.39	172	0.04	0.15
19	m	3.10	470	252	90	3.61	181	0.98	-0.16
20	m	2.89	381	330	210	3.21	53	-0.41	-0.77
21	m	2.46	615	265	423	3.40	81	0.32	-0.80
22	m	2.53	570	180	240	3.40	116	-0.30	0.84
23	m	1.85	150	180	150	3.15	575	1.39	-0.13
24	m	2.21	570	270	510	3.11	128	-1.36	-0.46
25	m	2.55	420	750	270	3.21	435	-0.65	-0.41
26	m	2.87	240	150	120	3.09	89	-0.71	-0.50
27	m	3.38	210	272	180	3.82	900	-2.23	-0.65
28	m					3.09	124	-0.05	0.47
29	m					2.78	32	0.02	-0.11
30	m					3.44	138	-0.89	-1.33
31	m					3.59	179	0.04	0.37
32	m					2.64	28	0.95	1.62
33	m					3.59	550	-0.03	-1.33
34	m					3.86	900	-1.39	-1.33

Tab. S2: Linear regression estimates for sex-specific regional volume/thickness changes per year ($N_{♀♀}$=27; $N_{♂♂}$=32).

Analyzed brain region	Females	Males
Caudate nucleus (left)	-0.0373	-0.0189
Caudate nucleus (right)	-0.0535	-0.0272
Caudate nucleus (average)	-0.0454	-0.0230
Hippocampus (left)	0.0058	0.1940
Hippocampus (right)	0.0206	0.0119
Hippocampus (average)	0.0132	0.1030
Amygdala (left)	-0.0178	-0.0324
Amygdala (right)	-0.0203	-0.0039
Amygdala (average)	-0.0191	-0.0181
Intracranial	0.3562	-0.5483
Thalamus	-0.0664	-0.1568
Splenium	-0.0527	-0.0498
Septum	-0.0111	0.0119
cxSplenium	-0.0130	-0.0181
cx6	-0.0136	-0.0170
cx4	0.0002	-0.0104
cxFrontal	-0.0067	-0.0137
cx24	-0.0132	-0.0159
cx23	-0.0089	-0.0072
cxCingulum	-0.0110	-0.0115
cx5	-0.0091	-0.0141
cx7	-0.0102	-0.0145
cxParietal	-0.0097	-0.0143
cx21	-0.0130	-0.0218
cx22	-0.0061	-0.0016
cx28	0.0029	0.0015
cxTemporal	-0.0054	-0.0073
cx17	-0.0068	-0.0211
cx18	-0.0115	-0.0255
cxOccipital	-0.0092	-0.0233
cxTotalAverage	-0.0084	-0.0140
cx13	-0.0064	-0.0183

Tab. S3: Full overview of Spearman's correlation analyses for the cognitive experiments (N=21); delay-corrected and normalized data.
Significance levels (code:*p≤0.05; **p≤0.01; n.s.= no significance), Spearman's correlation coefficients (r_{SP}) and p-values (p_{SP}) are presented for: PD 80, PDR 50, PDR 50-80.

	**PD 80**			**PDR 50**			**PDR 50-80**		
**Analyzed brain region**	r_{SP}	p_{SP}	**Signif.**	r_{SP}	p_{SP}	**Signif.**	r_{SP}	p_{SP}	**Signif.**
Caudate nucleus (left)	-0.3103	0.1709	n.s.	0.1418	0.5397	n.s.	-0.1700	0.4613	n.s.
Caudate nucleus (right)	-0.2069	0.3682	n.s.	0.1399	0.5453	n.s.	-0.3563	0.1129	n.s.
Caudate nucleus (average)	-0.2297	0.3166	n.s.	0.1783	0.4394	n.s.	-0.3269	0.1480	n.s.
Hippocampus (left)	-0.4424	0.0446	*	-0.4053	0.0683	n.s.	-0.1399	0.5452	n.s.
Hippocampus (right)	-0.2245	0.3280	n.s.	0.0950	0.6821	n.s.	0.1484	0.5208	n.s.
Hippocampus (average)	-0.3754	0.0935	n.s.	-0.1926	0.4030	n.s.	-0.0432	0.8527	n.s.
Amygdala (left)	0.0195	0.9331	n.s.	-0.0696	0.7643	n.s.	0.0379	0.8704	n.s.
Amygdala (right)	-0.0950	0.6821	n.s.	-0.2062	0.3697	n.s.	-0.3151	0.1641	n.s.
Amygdala (average)	0.0085	0.9710	n.s.	-0.1340	0.5625	n.s.	-0.0948	0.6827	n.s.
Intracranial	-0.1620	0.4829	n.s.	-0.2902	0.2020	n.s.	-0.1229	0.5955	n.s.
Thalamus	-0.2219	0.3338	n.s.	-0.6389	0.0018	**	-0.0948	0.6827	n.s.
Splenium	-0.1464	0.5266	n.s.	-0.2238	0.3294	n.s.	-0.1890	0.4120	n.s.
Septum	0.0390	0.8666	n.s.	0.3533	0.1162	n.s.	-0.0150	0.9484	n.s.
cxSplenium	-0.1269	0.5837	n.s.	0.0481	0.8358	n.s.	-0.0445	0.8482	n.s.
cx6	-0.2115	0.3575	n.s.	0.0878	0.7050	n.s.	-0.0909	0.6952	n.s.
cx4	0.1034	0.6554	n.s.	0.4079	0.0664	n.s.	0.0739	0.7503	n.s.
cxFrontal	-0.0631	0.7858	n.s.	0.2902	0.2020	n.s.	-0.0072	0.9753	n.s.
cx24	0.2030	0.3775	n.s.	0.0696	0.7643	n.s.	0.4949	0.0225	*
cx23	0.0202	0.9308	n.s.	-0.2212	0.3352	n.s.	-0.0824	0.7226	n.s.
cxCingulum	0.1516	0.5118	n.s.	-0.0573	0.8053	n.s.	0.2962	0.1924	n.s.
cx5	0.2095	0.3621	n.s.	0.1692	0.4635	n.s.	0.0974	0.6744	n.s.
cx7	0.0651	0.7793	n.s.	-0.0605	0.7944	n.s.	0.1040	0.6538	n.s.
cxParietal	0.1034	0.6554	n.s.	0.0481	0.8358	n.s.	0.0484	0.8350	n.s.
cx21	0.1646	0.4758	n.s.	0.3416	0.1297	n.s.	0.1399	0.5452	n.s.
cx22	0.3943	0.0770	n.s.	0.3643	0.1044	n.s.	0.3635	0.1053	n.s.
cx28	0.3513	0.1184	n.s.	-0.0696	0.7643	n.s.	0.1020	0.6600	n.s.
cxTemporal	0.4522	0.0396	*	0.3383	0.1336	n.s.	0.3119	0.1687	n.s.
cx17	0.0937	0.6863	n.s.	0.2928	0.1978	n.s.	0.0909	0.6952	n.s.
cx18	-0.0494	0.8314	n.s.	0.1165	0.6152	n.s.	-0.0889	0.7015	n.s.
cxOccipital	-0.0553	0.8118	n.s.	0.2674	0.2413	n.s.	0.0052	0.9820	n.s.
cxTotalAverage	0.0781	0.7366	n.s.	0.2082	0.3651	n.s.	0.1458	0.5283	n.s.
cx13	0.0306	0.8953	n.s.	0.0586	0.8009	n.s.	-0.0582	0.8022	n.s.

Tab. S4: Full overview of Spearman's correlation analyses for the open-field based experiments (N=30); delay-corrected and normalized data. Significance levels (code:*p≤0.05; **p≤0.01; n.s.= no significance), Spearman's correlation coefficients (r_{SP}) and p-values (p_{SP}) are presented for: OF 1, NO 1, SBE.

	**SBE**			**OF 1**			**NO 1**		
**Analyzed brain region**	**r_{SP}**	**p_{SP}**	**Signif.**	**r_{SP}**	**p_{SP}**	**Signif.**	**r_{SP}**	**p_{SP}**	**Signif.**
Caudate nucleus (left)	-0.2312	0.2190	n.s.	-0.0914	0.6296	n.s.	-0.3998	0.0286	*
Caudate nucleus (right)	-0.1022	0.5909	n.s.	-0.0314	0.8694	n.s.	-0.2080	0.2700	n.s.
Caudate nucleus (average)	-0.1831	0.3328	n.s.	-0.0848	0.6550	n.s.	-0.3218	0.0829	n.s.
Hippocampus (left)	-0.1450	0.4446	n.s.	-0.1168	0.5373	n.s.	-0.0399	0.8343	n.s.
Hippocampus (right)	-0.2539	0.1757	n.s.	-0.1106	0.5593	n.s.	-0.0002	0.9991	n.s.
Hippocampus (average)	-0.2063	0.2742	n.s.	-0.0861	0.6499	n.s.	0.0029	0.9879	n.s.
Amygdala (left)	-0.4907	0.0059	**	0.3112	0.0944	n.s.	0.2114	0.2622	n.s.
Amygdala (right)	-0.3479	0.0596	n.s.	0.1800	0.3398	n.s.	0.0392	0.8371	n.s.
Amygdala (average)	-0.4769	0.0077	**	0.2836	0.1287	n.s.	0.1394	0.4625	n.s.
Intracranial	0.2154	0.2530	n.s.	0.1479	0.4336	n.s.	-0.0027	0.9888	n.s.
Thalamus	-0.2622	0.1616	n.s.	0.1030	0.5867	n.s.	-0.0608	0.7496	n.s.
Splenium	-0.1060	0.5771	n.s.	0.1519	0.4212	n.s.	-0.1882	0.3193	n.s.
Septum	0.0846	0.6565	n.s.	0.0576	0.7618	n.s.	0.3679	0.0455	*
cxSplenium	-0.0976	0.6080	n.s.	0.0331	0.8620	n.s.	-0.2087	0.2684	n.s.
cx6	-0.1913	0.3111	n.s.	-0.4652	0.0103	*	-0.0891	0.6397	n.s.
cx4	-0.1247	0.5113	n.s.	-0.2538	0.1753	n.s.	-0.0849	0.6557	n.s.
cxFrontal	-0.1924	0.3083	n.s.	-0.3740	0.0425	*	-0.1258	0.5076	n.s.
cx24	0.1000	0.5990	n.s.	-0.4661	0.0101	*	-0.3508	0.0574	n.s.
cx23	-0.1071	0.5731	n.s.	0.0269	0.8879	n.s.	0.0220	0.9079	n.s.
cxCingulum	0.0552	0.7719	n.s.	-0.3103	0.0954	n.s.	-0.2223	0.2378	n.s.
cx5	-0.2853	0.1264	n.s.	-0.2369	0.2066	n.s.	-0.1330	0.4837	n.s.
cx7	-0.1091	0.5659	n.s.	-0.4082	0.0260	*	-0.1822	0.3353	n.s.
cxParietal	-0.2348	0.2117	n.s.	-0.3295	0.0759	n.s.	-0.1327	0.4844	n.s.
cx21	-0.1218	0.5213	n.s.	-0.1706	0.3657	n.s.	-0.1243	0.5129	n.s.
cx22	0.0272	0.8867	n.s.	-0.0661	0.7279	n.s.	-0.0987	0.6040	n.s.
cx28	-0.2666	0.1544	n.s.	0.0665	0.7261	n.s.	-0.0808	0.6711	n.s.
cxTemporal	-0.1708	0.3667	n.s.	-0.0407	0.8307	n.s.	-0.1196	0.5290	n.s.
cx17	-0.1506	0.4271	n.s.	-0.2245	0.2321	n.s.	-0.0510	0.7890	n.s.
cx18	-0.2366	0.2082	n.s.	-0.1488	0.4309	n.s.	-0.2608	0.1639	n.s.
cxOccipital	-0.2348	0.2117	n.s.	-0.1533	0.4171	n.s.	-0.2082	0.2695	n.s.
cxTotalAverage	-0.1746	0.3560	n.s.	-0.3366	0.0695	n.s.	-0.2218	0.2388	n.s.
cx13	-0.1223	0.5197	n.s.	0.0576	0.7618	n.s.	-0.0296	0.8765	n.s.

Tab. S5: Full overview of Spearman's correlation analyses for the cognitive experiments (N=21); non-corrected, normalized raw data. Significance levels (code: *p≤0.05; **p≤0.01; n.s.= no significance), Spearman's correlation coefficients (r_{SP}) and p-values (p_{SP}) are presented for: PD 80, PDR 50, PDR 50-80.

	**PD 80**			**PDR 50**			**PDR 50-80**		
**Analyzed brain region**	r_{SP}	p_{SP}	**Signif.**	r_{SP}	p_{SP}	**Signif.**	r_{SP}	p_{SP}	**Signif.**
Caudate nucleus (left)	-0.3071	0.1757	n.s.	0.1711	0.4583	n.s.	-0.1582	0.4933	n.s.
Caudate nucleus (right)	-0.1438	0.5341	n.s.	0.1861	0.4193	n.s.	-0.3367	0.1356	n.s.
Caudate nucleus (average)	-0.2433	0.2878	n.s.	0.1509	0.5137	n.s.	-0.2570	0.2608	n.s.
Hippocampus (left)	-0.3221	0.1545	n.s.	-0.3897	0.0807	n.s.	-0.0347	0.8815	n.s.
Hippocampus (right)	-0.2459	0.2825	n.s.	0.0859	0.7113	n.s.	0.1281	0.5799	n.s.
Hippocampus (average)	-0.3227	0.1536	n.s.	-0.1978	0.3901	n.s.	0.0327	0.8881	n.s.
Amygdala (left)	0.0241	0.9175	n.s.	-0.0833	0.7197	n.s.	0.0314	0.8926	n.s.
Amygdala (right)	-0.0540	0.8162	n.s.	-0.1984	0.3885	n.s.	-0.2779	0.2226	n.s.
Amygdala (average)	0.0085	0.9710	n.s.	-0.1340	0.5625	n.s.	-0.0948	0.6827	n.s.
Intracranial	-0.1971	0.3917	n.s.	-0.3006	0.1855	n.s.	-0.1863	0.4187	n.s.
Thalamus	-0.2433	0.2878	n.s.	-0.6610	0.0011	**	-0.1301	0.5740	n.s.
Splenium	-0.1555	0.5009	n.s.	-0.2342	0.3068	n.s.	-0.1929	0.4022	n.s.
Septum	0.0839	0.7176	n.s.	0.3149	0.1644	n.s.	0.0235	0.9193	n.s.
cxSplenium	-0.1815	0.4310	n.s.	0.0488	0.8336	n.s.	-0.0889	0.7015	n.s.
cx6	-0.2062	0.3697	n.s.	0.0735	0.7515	n.s.	-0.0948	0.6827	n.s.
cx4	0.0384	0.8688	n.s.	0.4157	0.0609	n.s.	0.0092	0.9686	n.s.
cxFrontal	-0.0761	0.7429	n.s.	0.2856	0.2094	n.s.	-0.0320	0.8904	n.s.
cx24	0.1327	0.5663	n.s.	0.0742	0.7493	n.s.	0.5067	0.0191	*
cx23	0.0104	0.9643	n.s.	-0.2420	0.2905	n.s.	-0.0667	0.7739	n.s.
cxCingulum	0.1178	0.6112	n.s.	-0.0455	0.8446	n.s.	0.2883	0.2050	n.s.
cx5	0.1809	0.4327	n.s.	0.1750	0.4480	n.s.	0.0556	0.8109	n.s.
cx7	0.0228	0.9220	n.s.	-0.0384	0.8688	n.s.	0.0490	0.8328	n.s.
cxParietal	0.0852	0.7134	n.s.	0.0520	0.8227	n.s.	0.0628	0.7869	n.s.
cx21	0.0169	0.9420	n.s.	0.3591	0.1098	n.s.	0.0582	0.8022	n.s.
cx22	0.4105	0.0645	n.s.	0.3513	0.1184	n.s.	0.3655	0.1033	n.s.
cx28	0.3481	0.1220	n.s.	-0.0774	0.7387	n.s.	0.0843	0.7162	n.s.
cxTemporal	0.4255	0.0545	n.s.	0.3175	0.1608	n.s.	0.2674	0.2412	n.s.
cx17	0.0534	0.8183	n.s.	0.3683	0.1005	n.s.	0.0517	0.8240	n.s.
cx18	-0.1269	0.5837	n.s.	0.1646	0.4758	n.s.	-0.1347	0.5605	n.s.
cxOccipital	-0.1366	0.5548	n.s.	0.3032	0.1815	n.s.	-0.1419	0.5396	n.s.
cxTotalAverage	0.0852	0.7134	n.s.	0.2199	0.3381	n.s.	0.1170	0.6134	n.s.
cx13	0.0124	0.9576	n.s.	0.0442	0.8490	n.s.	-0.1020	0.6600	n.s.

Tab. S6: Full overview of Spearman's correlation analyses for the open-field based experiments (N=30); non-corrected, normalized raw data. Significance levels (code:*p≤0.05; **p≤0.01; n.s.= no significance), Spearman's correlation coefficients (r_{SP}) and p-values (p_{SP}) are presented for: OF 1, NO 1, SBE.

	**SBE**			**OF 1**			**NO 1**		
**Analyzed brain region**	r_{SP}	p_{SP}	**Signif.**	r_{SP}	p_{SP}	**Signif.**	r_{SP}	p_{SP}	**Signif.**
Caudate nucleus (left)	-0.2357	0.2100	n.s.	-0.0808	0.6704	n.s.	-0.3962	0.0302	*
Caudate nucleus (right)	-0.1365	0.4719	n.s.	-0.0456	0.8106	n.s.	-0.1753	0.3542	n.s.
Caudate nucleus (average)	-0.1896	0.3157	n.s.	-0.0808	0.6704	n.s.	-0.3205	0.0842	n.s.
Hippocampus (left)	-0.1276	0.5015	n.s.	-0.2196	0.2426	n.s.	-0.1443	0.4467	n.s.
Hippocampus (right)	-0.2499	0.1829	n.s.	-0.1123	0.5530	n.s.	-0.0071	0.9702	n.s.
Hippocampus (average)	-0.2263	0.2292	n.s.	-0.1524	0.4198	n.s.	-0.0394	0.8361	n.s.
Amygdala (left)	-0.5023	0.0047	**	0.3281	0.0771	n.s.	0.2352	0.2109	n.s.
Amygdala (right)	-0.3537	0.0552	n.s.	0.1849	0.3266	n.s.	0.0392	0.8371	n.s.
Amygdala (average)	-0.4960	0.0053	**	0.3028	0.1040	n.s.	0.1715	0.3649	n.s.
Intracranial	0.2294	0.2226	n.s.	0.1697	0.3683	n.s.	0.0249	0.8959	n.s.
Thalamus	-0.2296	0.2222	n.s.	0.1648	0.3824	n.s.	0.0243	0.8987	n.s.
Splenium	-0.1232	0.5167	n.s.	0.1782	0.3446	n.s.	-0.1399	0.4610	n.s.
Septum	0.1138	0.5492	n.s.	0.0140	0.9420	n.s.	0.3111	0.0942	n.s.
cxSplenium	-0.1506	0.4271	n.s.	0.0861	0.6499	n.s.	-0.1290	0.4971	n.s.
cx6	-0.2105	0.2642	n.s.	-0.4331	0.0176	*	-0.0532	0.7800	n.s.
cx4	-0.1207	0.5251	n.s.	-0.2174	0.2475	n.s.	-0.0316	0.8682	n.s.
cxFrontal	-0.2361	0.2091	n.s.	-0.2917	0.1179	n.s.	-0.0007	0.9972	n.s.
cx24	0.0067	0.9720	n.s.	-0.4198	0.0217	*	-0.2786	0.1360	n.s.
cx23	-0.1466	0.4396	n.s.	0.0772	0.6842	n.s.	0.0713	0.7082	n.s.
cxCingulum	0.0004	0.9981	n.s.	-0.2347	0.2110	n.s.	-0.1102	0.5619	n.s.
cx5	-0.3270	0.0778	n.s.	-0.1996	0.2891	n.s.	-0.0617	0.7460	n.s.
cx7	-0.1437	0.4488	n.s.	-0.3326	0.0730	n.s.	-0.1332	0.4829	n.s.
cxParietal	-0.2354	0.2104	n.s.	-0.2850	0.1268	n.s.	-0.0882	0.6430	n.s.
cx21	-0.2085	0.2689	n.s.	-0.0830	0.6618	n.s.	0.0102	0.9571	n.s.
cx22	0.0078	0.9674	n.s.	-0.0452	0.8124	n.s.	-0.0898	0.6372	n.s.
cx28	-0.2568	0.1707	n.s.	0.0607	0.7492	n.s.	-0.1029	0.5885	n.s.
cxTemporal	-0.1980	0.2942	n.s.	-0.0416	0.8270	n.s.	-0.0920	0.6288	n.s.
cx17	-0.1793	0.3431	n.s.	-0.1382	0.4650	n.s.	0.0383	0.8407	n.s.
cx18	-0.3101	0.0954	n.s.	0.0274	0.8861	n.s.	-0.1129	0.5524	n.s.
cxOccipital	-0.3312	0.0738	n.s.	-0.0314	0.8694	n.s.	-0.0428	0.8225	n.s.
cxTotalAverage	-0.2807	0.1330	n.s.	-0.2538	0.1753	n.s.	-0.0929	0.6255	n.s.
cx13	-0.1713	0.3654	n.s.	0.1293	0.4944	n.s.	0.0497	0.7944	n.s.
